# Clinical characteristics of anti-SAE antibodies in Chinese patients with dermatomyositis in comparison with different patient cohorts

**DOI:** 10.1038/s41598-017-00240-6

**Published:** 2017-03-15

**Authors:** Yongpeng Ge, Xin Lu, Xiaoming Shu, Qinglin Peng, Guochun Wang

**Affiliations:** Department of Rheumatology, China-Japan Friendship Hospital, Haoyang District Beijing, China

## Abstract

This study aimed to analyze the clinical features of anti-SAE antibodies in Chinese myositis patients in comparison with different cohorts. The anti-SAE antibodies were tested in myositis patients and in control subjects. Long-term follow-up was conducted on the antibody-positive patients. Anti-SAE antibodies were exclusively present in 12 out of 394 (3.0%) adult dermatomyositis (DM) patients. Of the anti-SAE-positive DM patients, 75% had distinctive diffuse dark-red or pigment-like skin rashes, and 67% of these patients experienced mild muscle weakness. Muscular biopsies showed mild pathological manifestations. Compared with the antibody-negative group, the average age of dermatomyositis onset in the antibody-positive group was higher, and dysphagia occurred more frequently noted (*p* = 0.012). Only 9 patients received follow-up, 7 experienced improvement after treatment. The anti-SAE antibody levels correlated with improved disease condition. The anti-SAE antibody was found exclusively in adult DM patients, occurring infrequently in Chinese patients. In addition to a diffuse dark-red or pigment-like skin rash and mild muscular weakness, common symptoms included propensity for developing dysphagia. Serum levels of the anti-SAE antibody correlated with myositis disease activity, and anti-SAE-positive patients were responsive to treatment.

## Introduction

Idiopathic inflammatory myopathies (IIMs) are a group of clinically heterogeneous, autoimmune inflammatory muscular disorders characterized by muscular weakness and multisystem involvement. The main clinical subtypes of IIMs among Chinese patient populations are polymyositis (PM) and dermatomyositis (DM), including clinical amyopathic dermatomyositis (CADM), and juvenile dermatomyositis (JDM). IIM autoantibodies can be grouped into myositis-specific autoantibodies (MSAs) and myositis-associated autoantibodies (MAAs). The presence of MSAs and MAAs has not only become a key factor in classifying and diagnosing IIMs, but it is also increasingly used to define clinical subsets of patients.

Among the MSAs, the cytoplasmic enzyme aminoacyl-tRNA synthetase (ARS), the nuclear helicase protein Mi-2, and the cytoplasmic complex signal recognition particle (SRP) have been well studied. Recently, several novel antibodies have been described as being associated with distinct myositis subsets, potentially further expanding the MSA panel^1, 2^. Anti-small ubiquitin-like modifier activating enzyme (anti-SAE) antibodies were first identified by Betteridge *et al*. in 2007^3^, and the same group then confirmed and characterized these autoantibodies in a larger cohort of IIM patients from the United Kingdom^4^. Anti-SAE antibodies were identified in 8% of adult patients with DM, a patient subset that is especially prone to severe skin disease and mild muscle involvement at disease onset. However, other studies have revealed that the antibody occurs infrequently in IIM patients, and that other clinical features are common^5–9^.

The prevalence of anti-SAE antibodies in Chinese IIM patients, the clinical features of Chinese patients carrying anti-SAE antibodies, and the differences between our study cohort and other study cohorts were previously unknown. To address these questions, we measured serum anti-SAE antibodies levels in 570 PM/DM patients and 90 controls, and compared the differences between the clinical features of the anti-SAE antibody-positive Chinese patients and those of the other cohorts that carried the anti-SAE antibody. The patients carrying anti-SAE antibodies also received follow-up to analyze how this subgroup responded to therapy, and whether anti-SAE antibody levels could predict disease activity or prognosis.

## Results

### The frequency of anti-SAE antibodies in Chinese patients with PM/DM

In total, 14 patients were discovered by enzyme-linked immuno sorbent assay (ELISA) to be carrying anti-SAE1 antibodies, and 12 of these cases were confirmed by protein immunoprecipitation (IPP). The overall frequency of anti-SAE antibody occurrence in the PM/DM cohort was 2.1%. Anti-SAE antibodies were found exclusively in patients with adult-onset DM (n = 394), with a frequency of 3.0%. Among the 144 PM patients, none carried anti-SAE antibodies, and none of the 32 JDM patients carried the antibody. Anti-SAE antibodies were not present in the healthy controls or in the disease control groups, i.e. patients suffering from idiopathic hyper-creatine kinase-emia, systemic sclerosis (SSc), muscular dystrophy, myasthenia gravis, motor neuron disease, or mitochondrial myopathy.

### Characteristics of anti-SAE antibody-positive Chinese DM patients

There were 9 (75%) females and 3 (25%) males among the 12 patients carrying the anti-SAE antibody (Table 1). The clinical features of the patients are listed in Table 1. The average age of DM onset among the anti-SAE antibody-positive patients was 59.1 years. Of these patients, 9/12 (75%) presented with the hallmark cutaneous manifestations of DM (heliotrope sign rash and Gottron’s sign rash), and 6/12 (50%) showed shawl sign rash and V sign rash. In addition to these classic skin rashes, another characteristic skin lesion is a diffuse dark-red skin rash or diffuse pigment-like skin rash observed in 75% (9/12) of patients carrying the anti-SAE antibody. Rash ulceration appeared in 7/12 (58%) patients.

**Table 1:** Clinical features of DM patients with anti-SAE antibodies.

	Patient											
Features	1	2	3	4	5	6	7	8	9	10	11	12
Onset age	82	48	58	48	54	73	45	53	57	55	68	68
Age at diagnosis	82	48	58	48	55	73	45	67	57	55	68	69
Follow up time (months)	36	8	21	12	40	30	NA	8	21	NA	NA	2.5
Gender	F	M	F	M	F	F	F	F	F	F	F	M
Heliotrope sign	Y	Y	Y	Y	Y	Y	N	N	N	Y	Y	Y
V sign	N	Y	Y	N	Y	Y	N	N	N	Y	Y	N
Gottron’s sign	Y	N	Y	Y	Y	N	Y	Y	Y	N	Y	Y
Shawl sign	Y	Y	Y	N	N	Y	Y	N	N	N	Y	N
Mechanic’s hands	Y	N	N	N	N	Y	Y	Y	Y	N	N	Y
Diffuse skin rash	Y	Y	Y	Y	Y	N	N	Y	N	Y	Y	Y
Skin ulceration	Y	Y	Y	Y	Y	N	N	N	N	Y	Y	N
Muscular weakness	Y	Y	Y	Y	N	Y	Y	Y	N	N	N	Y
Myalgia	N	Y	N	N	N	Y	Y	Y	N	NA	N	Y
Arthralgia	Y	N	N	N	Y	N	N	Y	Y	NA	N	N
CK (IU/L)	650	1282	N	361	N	N	N	314	592	NA	N	251
Dysphagia	Y	Y	N	Y	N	Y	N	Y	N	NA	Y	Y
PAH	N	Y	NA	NA	N	N	NA	Y	N	NA	N	Y
ILD	N	Y	Y	Y	N	N	Y	Y	Y	NA	N	Y
Cancer	Y	N	N	N	N	N	N	Y	N	NA	N	N
MSAs	TIF1	N	PL-7	N	N	N	Jo-1	N	Jo-1	N	N	N
ESR (mm/h)	36	4	28	45	7	62	6	NA	60	NA	21	62
CRP (mg/dl)	7.39	1.01	3.36	0.17	0.22	NA	0.19	0.59	1.95	NA	0.15	2.4
Presentation	S	S	S	S/M	S	NA	NA	S/M	S/M	NA	S	S

NA, not available; CK, creatine kinase; PAH, pulmonary arterial hypertension; ILD, interstitial lung disease; CRP, C-reactive protein; ESR, erythrocyte sedimentation rate; MSAs, myositis-specific autoantibodies; MAAs, myositis-associated autoantibodies; S, initially presented with skin disease only; S/M, presented with skin and muscular disease.

Six patients carrying anti-SAE antibodies exhibited cutaneous DM only (without muscular weakness), but then gradually developed typical myositis. The median time interval from initial skin rash to myositis onset was 3.5 months.

According to available data on these patients (complete data for one patient was unavailable), 8/12 (67%) patients exhibited muscular weakness; 6/12 (50%) experienced mechanic’s hands; 5/11 (45%) patients suffered myalgia; and 4/11 (36%) suffered arthralgia.

Seven out of eleven patients (64%) were diagnosed with interstitial lung disease (ILD) via lung imaging examination, but only one had respiratory symptoms. Seven (64%) patients experienced dysphagia; five patients experienced difficulty in digesting solid food; four patients coughed when drinking water; and three patients had difficulty speaking coherently. Nasogastric tubes were applied in two of the cases.

Three out of eight (38%) patients experienced the complication of mild pulmonary arterial hypertension (PAH). Cancers were observed in two patients. Eight out of 12 (67%) patients were ANA-positive. Five patients tested positive for other MAAs, including anti-RO-52, anti-ribonucleoproteins (RNPs), and the anticardiolipin (ACL) antibody. Other MSAs found in these patients include: transcription intermediary factor 1 gamma (TIF1-γ), PL-7, and anti-Jo-1.

### Pathological Features of Muscles

**Figure 1:**
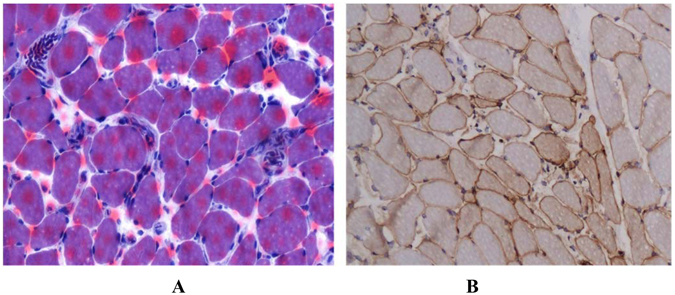
Histologic and immunohistologic analysis of muscle biopsies from the biceps of an anti-SAE antibody-positive patient. (**A**) Stain with HE (**B**) stain with anti-MHC-I antibody.

Muscular biopsies were available for seven patients carrying the anti-SAE antibody. Pathological lesions in muscular tissues were comparatively mild in most patients. Six (86%) muscular biopsies did not show degeneration, necrosis, atrophy, or regeneration of muscle fiber. They displayed small blood vessels in muscle fibers and perimysium infiltrated with small inflammatory cells (Fig. 1). Only one patient suffered from a bit of myofiber necrosis and a few inflammatory cells concomitantly.

### The clinical difference between anti-SAE antibody-positive DM patients and anti-SAE antibody-negative DM patients

The average age of DM onset among anti-SAE antibody-positive patients was significantly older than DM onset age among anti-SAE antibody-negative patients (*p* < 0.05). The occurrence of skin rashes – such as Heliotrope sign, V sign, Shawl sign, Gottron’s sign, and Mechanic’s hands – in the anti-SAE antibody-positive group was not significantly different from occurrence in the antibody-negative group (*p* > 0.05). Eight out of the 12 anti-SAE antibody-positive patients experienced muscular weakness, but most of the serum muscle enzyme levels were normal or slightly elevated. Levels of serum muscle enzymes – including creatine kinase (CK), lactate dehydrogenase (LDH) and hydroxybutyric acid dehydrogenase (HBDH) – in anti-SAE antibody-positive patients were not significantly lower than those in antibody-negative patients (*p* > 0.05 for all enzymes). As for involvement of internal organs, the frequency of dysphagia was significantly higher in antibody-positive patients than in antibody-negative patients (64% vs 25%, *p* = 0.012), and the incidences of ILD and cancer were similar in both patient types (both *p* values > 0.05). Although ferritin, immunoglobulins A, G, M (IgA, IgG, IgM), and C-reactive protein (CRP) levels as well as erythrocyte sedimentation rate (ESR) in anti-SAE antibody-positive patients were all lower than those in the antibody-negative group, the differences were not statistically significant (Table 2).

**Table 2:** Differences between anti-SAE antibody-positive group and antibody–negative group.

Characteristic	Anti-SAE antibody-positive patients	Anti-SAE antibody-negative patients
N	12	382
Female:Male	9:3	277:105
Age (years)	59.1 ± 11.3	48.6 ± 14.0*
Heliotrope sign	9/12 (75%)	213/338 (63%)
V sign	6/12 (50%)	184/347 (53%)
Shawl sign	6/12 (50%)	154/348 (44%)
Gottron’s sign	9/12 (75%)	244/344 (71%)
Mechanic’s hands	6/12 (50%)	87/334 (26%)
Muscular weakness	8/12 (67%)	153/267 (57%)
Dysphagia	7/11 (64%)	82/328* (25%)
Arthralgia	4/11 (36%)	114/301 (38%)
ILD	7/11 (64%)	150/332 (45%)
Cancer	2/11 (18%)	27/257 (11%)
CK (IU/L)	286.6 ± 368.2	691.6 ± 1488.4
LDH (IU/L)	254.9 ± 62.3	373.0 ± 338.0
HBDH (IU/L)	162.5 ± 42.2	274.8 ± 254.0
Ferritin (ng/ml)	360.6 ± 347.2	883.8 ± 2347.4
IgA (mg/dl)	209.8 ± 154.2	243.7 ± 121.8
IgG (mg/dl)	1112.3 ± 539.0	1296.1 ± 585.2
IgM (mg/dl)	86.9 ± 41.6	211.4 ± 831.7
C3 (mg/dl)	90.1 ± 25.0	88.2 ± 21.7
C4 (mg/dl)	22.3 ± 3.7	21.2 ± 8.5
CRP (mg/dl)	1.7 ± 2.3	2.0 ± 6.9
ESR (mm/h)	30.0 ± 21.2	50.1 ± 165.7

LDH, lactate dehydrogenase; HBDH, hydroxybutyric acid dehydrogenase; C3, complement 3; C4, complement 4; Ig, immunoglobulin; *p < 0.05.

### Features of anti-SAE occurrence in different myositis cohorts

#### Related studies

We found a total of seven related articles^3–9^, including four studies involving European populations, and three Japanese studies (Table 3). In total, there were 22 anti-SAE antibody-positive patients with DM among the four European studies, and 14 DM patients carrying anti-SAE antibodies were detected in the three Japanese studies, which included one JDM patient. Combined with our study subjects, there were a total of 25 adult Asian DM patients who carried the anti-SAE antibody. Including our study, a total of 1276 adult DM patients were tested for the presence of the anti-SAE antibody, and 47 (3.7%) patients were found to be carriers. Including the JDM case, the frequence of anti-SAE antibody occurrence was 3.5% among DM patients (48/1360).

**Table 3:** Frequency of anti-SAE occurrence in different myositis groups.

	Western cohorts				Asian cohorts		
Study	Betteridge	Tarricone	Betteridge	Bodoki	Fujimoto	Muro	Our study
	[3]	[5]	[4]	[8]	[6]	[7, 9]	
IIM	44	130	266	337	518	150	570
Adult DM	2/20 (10%)	5/75 (7%)	11/131 (8%)	4/73 (5%)	6/445 (1%)	7/138 (5%)	12/394 (3%)
PM	0/24	0/43	0/124	0/211	0/62	0	0/144
JDM	0	0/12	NA	0/17	1/11	0/12	0/32
F/M	1/1	NA	7/4	2/2	4/3	3/4	9/3
Heliotrope sign	2/2 (100%)	2/5 (40%)	9/11 (82%)	3/4 (75%)	4/7 (57%)	3/7 (43%)	9/12 (75%)
V sign	2/2 (100%)	NA	3/7 (43%)	1/4 (25%)	4/7 (57%)	5/7 (71%)	6/12 (50%)
Shawl sign	2/2 (100)	NA	3/7 (43%)	1/4 (25%)	6/7 (86%)	4/7 (57%)	6/12 (50%)
Gottron’s sign	2/2 (100%)	5/5 (100%)	9/11 (82%)	3/4 (75%)	6/7 (86%)	7/7 (100%)	9/12 (75%)
Muscular weakness	0/2 (0)	5/5 (100%)	11/11 (100%)	4/4 (100%)	6/7 (86%)	5/7 (71%)	8/12 (67%)
Periungal changes	2/2 (100%)	1/5 (20%)	8/8 (100%)	1/4 (25%)	5/5 (100%)	5/6 (83%)	NA
Arthralgia	NA	0/4 (0)	2/11 (18%)	2/4 (50%)	1/7 (14%)	0/7 (0)	4/11936%)
Elevated CK	2/2 (100%)	4/5 (80%)	9/11 (82%)	NA	5/7 (71%)	7/7 (100%)	6/11 (55%)
Dysphagia	2/2 (100%)	0/5 (0)	7/9 (78%)	3/4 (75%)	2/7 (29%)	3/7 (43%)	7/11 (64%)
ILD	2/2 (100%)	0/5 (0)	2/11 (18%)	1/4 (25%)	5/7 (71%)	5/7 (71%)	7/11 (64%)
Cancer	0/2 (0)	1/5 (20%)	2/11 (18%)	1/4 (25%)	1/7 (14%)	4/7 (57%)	2/11 (18%)

Among these anti-SAE antibody-positive DM patients, 41 (85%) presented with Gottron’s sign; 22/30 (73%) experienced periungal changes; 32/48 (67%) experienced heliotrope rash; 22/39 (56%) exhibit the shawl sign; and 21/39 (54%) exhibited the V sign. Regarding internal organ involvement, 24/45 (53%) suffered from dysphagia; 22/47 (47%) experienced ILD complications; and 11/47 (23%) were diagnosed with cancer.

#### Comparison between Chinese and Japanese cohorts

The occurrence of anti-SAE antibodies in adult Chinese DM patients seemed higher than in the Japanese patient group (3.0% vs 2.2%), but this difference was not statistically significant (*p* > 0.05).

The number of anti-SAE antibody-positive patients presenting with Heliotrope sign rash, V sign rash, shawl sign rash, Gottron’s sign rash, muscular weakness, and arthralgia was not statistically different between the Chinese and Japanese cohorts (*p* > 0.05). In the three Japanese studies, 5 patients had cancer, 10 had ILD, and 5 had dysphagia. However, these numbers were also not significantly different from those of the present study (all *p* > 0.05).

#### Follow-up study of anti-SAE antibody-positive patients

Among anti-SAE positive patients, 9 patients were received long-term monitoring. The follow-up time ranged from 2.5 months to 3.5 years (median 21 months). Anti-SAE antibody levels; serum CK levels; Manual Muscle Testing 8 (MMT-8), physician’s global activity (PGA), and Modified Myositis Disease Activity Assessment Tool (MYOACT) scores; and glucocorticoid dosage were monitored at each visit.

**Figure 2:**
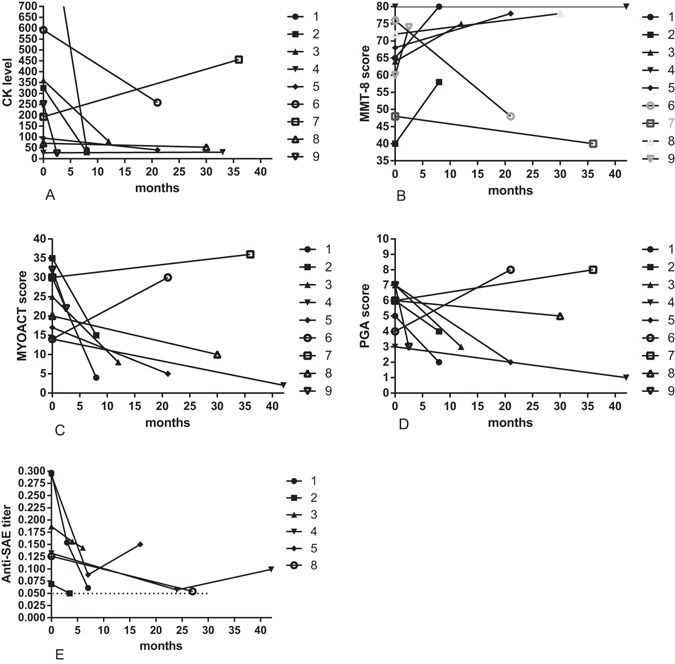
Follow-up findings from anti-SAE antibody-positive patients. CK levels (**A**), MMT-8 scores (**B**), MYOACT scores (**C**), PGA scores (**D**), and anti-SAE antibody levels (**E**) at baseline and after treatment.

Of these patients, seven (78%) responded well to glucocorticoid and/or immunosuppressive agents and experienced improvement in CK levels (Fig. 2A), MMT-8 scores (Fig. 2B), MYOACT scores (Fig. 2C), or PGA scores (Fig. 2D). The average glucocorticoid dosage was reduced from 61 mg/day to 25 mg/day for these patients. Furthermore, two patients regained normal muscular strength, normal CK levels, and normal skin appearance, and they suffered very little extramuscular disease activity. Two of these 9 patients (22%) did not experience improvement according to our improvement criteria. One of the two patients died of lung cancer before the last follow-up visit.

The data points representing anti-SAE antibody levels in the six patients who improved are shown in Fig. 2E. After treatment, anti-SAE antibody levels declined in all of these patients, and normalized in two (33%) of the six patients by the time their last follow-up visit occurred.

## Discussion

This is the first study to describe the prevalence of anti-SAE antibodies in a large sample of Chinese IIM patients. Anti-SAE antibodies were found exclusively in adult DM patients with a low rate of occurrence among patients tested for the antibody: an occurrence rate of 3.0%. Antibody-positive patients exhibited hallmark skin rashes and mild myopathy. These patients had late DM onset and experienced more dysphagia than did anti-SAE antibody-negative DM patients. Most of the antibody-positive patients responded well to immunosuppressive treatment and achieved a good prognosis. Therefore, serum levels of the anti-SAE antibody may correlate with disease activity.

The present study shows that the anti-SAE antibody has a very low prevalence among Chinese DM patients. These results are very similar to findings from the Japanese studies^6, 7, 9^, and significantly lower than those findings in the European studies^3–5, 8^. This difference in prevalence may be associated with ethnicity.

The main clinical features of muscular weakness and classic cutaneous manifestations – such as Heliotrope sign and Gottron’s sign – were observed in our cohort. We also found that another remarkable and distinguishing skin characteristic of anti-SAE antibody-positive DM patients was a diffuse dark-red skin rash or a diffuse pigment-like skin rash with concomitant rash ulceration. This feature was not mentioned by either the Japanese or European studies. However, dark-red or pigment-like skin rashes were not present exclusively in anti-SAE-positive patients; they also appeared in some anti-Mi-2 antibody-positive DM patients.

Although most of patients exhibited muscular weakness, their weakness was not severe. Only two patients’ muscular strength fell below grade 4 (equal to 64 points by MMT-8), according to the Medical Research Council (MRC) strength scale. Nearly half of the antibody-positive patients had normal CK levels. This finding was consistent with pathological findings that showed mild inflammation in muscular biopsies. In the present study, skin lesions occurred prior to muscular weakness in six patients. This phenomenon was also reported by Betteridge ZE *et al*. and Fujimoto M *et al*.^4, 6^. Furthermore, one patient who carried the anti-SAE antibody was diagnosed with CADM initially, but after 30 months of follow-up, muscular weakness occurred.

By comparing the clinical features of the anti-SAE-positive and -negative groups, we found the average DM onset age of the antibody-positive group was significantly greater than that of the antibody-negative group. This finding suggests that anti-SAE antibody-positive DM patients usually experience later-onset DM. Furthermore, we found that another prominent clinical feature of antibody-positive patients in our cohort was dysphagia. However, there were too few anti-SAE antibody-positive patients, so this potential association with dysphagia needs to be examined. In addition, our antibody-positive group had mean levels of serum muscle enzymes and ferritin that were lower than those in the antibody-negative group. This finding suggests that anti-SAE antibody-positive DM patients have lower disease activity than patients who do not carry the anti-SAE antibody. In the study by Betteridge Z *et al*., no significant clinical associations were observed in DM patients with and without anti-SAE antibodies, except for a higher frequency of periungal changes in the anti-SAE-positive group^4^.

In order to further understand the differences in clinical features between the different study ethnicities, we reviewed and compared our work with other published studies. The results showed that the incidence and clinical characteristics of the anti-SAE antibody-positive patients in our cohort did not differ significantly with those of the Japanese cohort. This finding may affirm the existence of genetic similarities between Asian DM patients. The incidence of ILD in DM patients carrying the anti-SAE antibody was remarkably high in both the Japanese cohort (73%) and our cohort (64%), and both incidence rates were much higher than that of the European cohort. This finding may indicate that anti-SAE antibody-positive DM patients from different cohorts are clinically heterogeneous, and Asian DM patients are prone to developing ILD.

One study from Japan showed that the incidence of cancer among DM patients carrying anti-SAE antibodies was significantly higher than in DM patients not carrying the antibody^9^. In the present study, two patients had been diagnosed with cancer, and the anti-TIF1γ antibody, a risk predictor for myositis-associated malignancy^10, 11^, was detected in one of them. However, larger sample sizes and longer follow-up periods are needed to demonstrate whether or not the presence of anti-SAE antibodies is a risk factor for malignancy in Chinese DM patients.

MSAs usually do not occur together. However, anti-SAE antibodies seem to coexist peacefully with other MSAs. In the present study, one-third of the anti-SAE antibody-positive patients also carried other MSAs simultaneously (one patient also carried anti-TIF1γ; two also carried anti-Jo-1; and one also carried anti-PL-7). Other studies also found that the anti-SAE antibody could co-exist with other MSAs^5, 9^. The reason for this phenomenon is not fully understood and requires further investigation. Other MSA (Jo-1/PL-7/TIF1γ) were detected by immunoblotting in our study. However, we couldn’t rule out the possibility of false positivity.

Nine anti-SAE positive patients received follow-up in this study. We found that most of them (78%) met the improvement criteria after they received glucocorticoid and/or immunosuppressive agent treatment. These results suggest that DM patients carrying anti-SAE antibodies may exhibit a subtype of treatment response well.

In order to understand whether the occurrence of anti-SAE antibody is associated with disease activity, we traced serial changes of antibody levels in six patients. We found that anti-SAE antibody levels decreased significantly in patients who improved. Furthermore, elevated antibody levels returned to normal titers after DM remission in two patients. In our cohort, antibody levels declined together with reduced CK levels, improved muscular strength, and improved overall condition in the follow-up period. Therefore, anti-SAE antibody titers may be a useful indicator of disease activity.

This study has several limitations. First, the study involves a relatively small number of patients, some of whom were not followed for a long enough period of time. Second, because this was a retrospective study, complete clinical data was not available for every patient. Lastly, some patients had already received immunotherapy before admission to our hospital; therefore, initial visit data do not reflect these patients’ conditions prior to treatment. The sample size is relatively small for such a rare antibody. A multicenter study would be helpful in systematically cataloguing the characteristics of anti-SAE-positive patients

In conclusion, the present study demonstrates that the anti-SAE antibody was found exclusively in adult DM patients and occurred infrequently among Chinese patients. The anti-SAE-positive subtype of patients exhibited a distinguishing diffuse dark-red or pigment-like skin rash, experienced mild muscular weakness, easily developed dysphagia, and responded well to immunosuppressive treatment.

## Methods and Materials

### Patients and sera

All patients (n = 570) met either the Bohan and Peter criteria or the European Neuromuscular Centre criteria (ENMC) for PM and DM^12, 13^: 144 of these patients had PM, and 426 patients had DM, including 26 with CADM and 32 with JDM. Serum samples were obtained between March 2005 and Dec 2015. The following data were obtained from their medical records: age; gender; medical history; muscular strength; pulmonary function (PF); electrocardiogram (ECG); ultrasonic cardiogram (UCG); chest computed tomography (CT) images; levels of CK; LDH and HBDH; tumor markers; presence of MAAs and other MSAs; levels of IgA, IgG, IgM; CRP; Complement 3 (C3) and Complement 4 (C4); as well as ESR. Muscular weakness was evaluated according to MMT-8 scores, the maximum score being 80^14^. Disease activity was evaluated by using the MYOACT^15^. Physician’s global activity assessment was evaluated by the visual analogue scale.

Patients who carried the anti-SAE antibody received follow-up, and their response to treatment was evaluated. Improvement was defined as an improvement of more than 20% on at least two of the four core parameters (MMT-8 score, MYOACT scores, PGA scores, and serum CK levels). The score of the other parameters (except for MMT-8) could decrease no more than 25%^15^.

Our study controls included blood from 40 healthy donors and from 50 patients with other inflammatory/autoimmune diseases, including 20 with idiopathic hyper-creatine kinase-emia, 10 with SSc, 5 with muscular dystrophy, 5 with myasthenia gravis, 5 with motor neuron disease, and 5 with hypothyroid myopathy. Basic data, including age and sex, were recorded for these control samples. Sera from all groups were collected and stored at −80 °C.

Specific binding of serum antibodies to recombinant SAE1 was analyzed using ELISA, which was performed as described in a previous study^8^. Briefly, wells of microtiter plates were coated with SAE1 protein (Origene) (50 ng/50 μl/well), diluted with PBS, and then blocked with 200 μl of a blocking buffer of 2% bovine serum albumin in PBS containing 0.05% Tween 20 (T-PBS) for 2 hours. Uncoated wells were used to measure the background levels of each sample. Sample sera diluted to a ratio of 1:500 with blocking buffer (50 μl/well) were incubated for 1 hour at room temperature, followed by incubation with anti-human IgG antibody conjugated with HRP (100 μl/well) at a 1:30,000 dilution after washing.

After incubation for 1 hour at room temperature, the plates were washed and incubated with Ultra TMB (100 μl/well) as the substrate, according to the manufacturer’s protocol. Optical density at 450 nm was determined. Each serum sample was tested in duplicate, and the mean subtracted background was used for data analysis. Positive results were greater than or equal to the mean (+5 standard deviations) of the units obtained from healthy control serum samples.

### Protein immunoprecipitation (IPP)

IPP from K562 cell extracts was performed according to protocols described by Fujimoto M *et al*.^10^. Briefly, a total of 10 ul of the patient’s serum was mixed with 2 mg of Protein A-Sepharose beads (GE Healthcare, UK) in 500 ul of IPP buffer (50 mM Tris, pH7.4, 150 mM NaCl, 1% NP-40) and incubated for 2 h at 4 °C,followed by washing 5 times with IPP buffer. IgG bound Protein A-Sepharose was then resuspended in 375 ul of IP buffer and incubated for 2 h at 4 °C with 125 ul K562 cell extracts derived from 5 × 10^6^ cells. The beads were then washed five times with IPP buffer and suspended in 5 × SDS sample buffer. After heating, immunoprecipitated proteins were fractionated by 10% SDS-polyacrylamide gel electrophoresis (PAGE), and then were electrophoretically transferred onto Immobilon-P (polyvinylidene difluoride) transfer membranes (Millipore, USA). Target proteins were detected by using the western blotting method with mouse monoclonal anti-human SAE1 (Abnova, China) antibodies.

### Standard protocol approvals and patient consents

All samples were obtained for research purposes. All participants provided signed informed consent for participation in this study. All the protocols were carried out in accordance with approved or provided guidelines. All patients’ data were anonymously used. This study was approved by the Research Review Committee (RRC) and the Ethical Review Committee (ERC) of the China-Japan Friendship Hospital.

### Literature review

In order to compare the difference between the different cohorts, relevant studies were pulled from databases such as PUBMED, EMBASE, and China National Knowledge Infrastructure (CNKI) between May 2006 and Feb 2016.

### Statistical analysis

All analyses were completed using SPSS 17.0, and *P* values less than 0.05 were considered significant. Correlation analysis was performed using the Spearman test. Quantitative variables are reported as means and were compared using a nonparametric test. Categorical variables are reported as numbers and/or percentages and were compared using the chi-square or, when appropriate, Fisher exact test.
